# The keys to happiness: Associations between personal values regarding core life domains and happiness in South Korea

**DOI:** 10.1371/journal.pone.0209821

**Published:** 2019-01-09

**Authors:** Min-Ah Lee, Ichiro Kawachi

**Affiliations:** 1 Department of Sociology, Chung-Ang University, Seoul, South Korea; 2 Department of Social and Behavioral Sciences, Harvard School of Public Health, Boston, Massachusetts, United States of America; Sogang University (South Korea), REPUBLIC OF KOREA

## Abstract

Personal values refer to the beliefs, principles or ideas that are important to people’s lives. We investigated the associations between personal values and happiness. We inquired about the importance of four different categories of personal values: prioritizing social relationships, extrinsic achievements, physical health, and spirituality. Data were drawn from the Korean General Social Survey (KGSS), a nationally representative cross-sectional sample collected over three years (i.e., 2007, 2008, and 2009). The findings showed that respondents prioritizing religion (i.e., spirituality) were the most likely to be happy, followed by those prioritizing social relationships, including family, friends, and neighbors. Those who prioritized extrinsic achievements (money, power, educational attainment, work, and leisure) as well as health were least likely to be happy. The findings suggest that pursuing goals focused on self-enhancement or self-centered value are less likely to result in happiness compared to pursuing alter-centered collective goals or self-transcendence/selflessness.

## Introduction

A growing literature has addressed the science of happiness, or subjective well-being (SWB). Although material well-being is a critical ingredient of human well-being, it has also been recognized that an increase in material well-being beyond a certain threshold (i.e. once basic wants have been satisfied) does not guarantee further increases in happiness [1–2] (although this point has also been debated [3]). This has influenced many scholars to seek other factors that determine subjective well-being [4–5].

In this context, a considerable number of studies have examined personal values, goals, or aspirations as important factors associated with subjective well-being [5–7]. Personal values may affect individuals’ daily lives as well as major decisions regarding their lives and futures, shaping their life trajectories, social relationships, and subjective well-being in the long run. For example, it is well known that holding intrinsic values, such as personal growth and affiliation, is positively associated with happiness, in contrast to holding extrinsic values, such as economic success and popularity [5, 8]. These studies clearly suggest that happiness is influenced by the personal values people hold in various life domains.

However, with the limitations of previous studies, questions remain regarding the association of personal values with subjective well-being. Although it is meaningful that previous studies have captured the relative propensity of individuals by using composite measures of personal values and goals [5, 8], less is known about whether and how personal values attached to specific life domains are associated with happiness. For example, are people who prioritize family happier than those who prioritize money? Is valuing religion more strongly associated with happiness than family? These questions motivated the current study to directly investigate how prioritizing specific life domains relates to happiness.

Recent studies have shown that prioritizing time more highly than money is positively associated with happiness [9–10]. Individuals may choose to allocate more of their time to making money, but often do so at the expense of neglecting social relationships (spending time with family, friends, and the community). The millionaire rapper and songwriter Sean “Diddy” Combs recently said in an interview that “I can always make more money, but I can’t make time”, which expresses the ideas that (a) investing in relationships does not cost money, but (b) making more money is often traded off against other uses of time. It has been discussed that prioritizing time over money is beneficial for happiness because it can improve the quality of social relationships [9–10]. Although a recent study has shown that prioritizing family over work and leisure results in higher life satisfaction [11], most studies have compared a limited number of contrasting domains (i.e., time vs money, family vs. work), but not included diverse life domains together. Valuing specific life domains, such as family, power, money, or religion, not only indicates personal values and attitudes toward life, but also affects individual behaviors and decision making.

Furthermore, most studies regarding personal values and happiness have been conducted in Western societies, with a few exceptions [8, 12], and have analyzed non-representative samples, such as convenience samples or samples of specific groups, such as college students [4, 8, 13]. It is therefore worth investigating these relationships using a representative sample in a non-Western societal setting such as South Korea. Korean society is traditionally founded on strong family-oriented values derived from Confucianism, although this has been weakening over the last several decades. In addition, religious influence on individual life might be stronger than other East Asian countries, although relatively weaker compared to other Western countries. As of 2015, it is reported that about 43.9% of Koreans have a religion. Among those who have a religion, 35.4% are Buddhists and 62.9% are Christians [14]. Among the total population, 15.5% are Buddhists while 27.7% are Christians [14]. This suggests that South Korea has a unique socio-cultural context in relation to Christianity and traditional values, which distinguishes it from other East Asian countries. For example, it is reported that only 1.5% of Japanese population are Christians as of 2012 [15]. South Korean society is therefore somewhat unique in the East Asian region for simultaneously maintaining Confucian family-oriented values together with Christianity.

In the current study we sought to investigate the effects of one’s personal values regarding core life domains on happiness. We used the Korean General Social Survey (KGSS) of a nationally representative sample, collected over three years (i.e., 2007, 2008, and 2009), which asked respondents to indicate their most valued life domain among the 10 presented, such as family and money, and to rate their happiness. We classified the personal values into four categories: prioritizing social relationships, extrinsic achievements, physical self, and spirituality. We begin with a literature review on the human value system and associations between personal values and subjective well-being.

## Literature review

### The structure and content of human values

Exploring the human value system can increase understanding of the content of personal values embedded in the system, which can be used to classify diverse life domains into common categories based on the nature of those human values. Schwartz [16–17] provided a two-dimensional circumplex model explaining the structure and content of human values. According to Schwartz [16–17], 10 types of values differentiated by motivational goals can be classified into four value dimensions: self-transcendence; self-enhancement; openness to change; conservation. Each type of value may conflict with other values if it is located in the opposite direction of the value dimension [16–17]. For example, self-transcendence, including universalism and benevolence, is opposite to self-enhancement, including achievement and power, while openness to change is opposite to conservation [17].

The contrast between self-enhancement and self-transcendence can be likened to the contrast between extrinsic and intrinsic values, although they are not synonymous. Intrinsic and extrinsic values are well-known descriptions of the content of human values and have been used to examine their associations with subjective well-being [12, 18]. Intrinsic values include personal growth, affiliation, community feeling, and physical health, whereas extrinsic values include financial success, image, and popularity, directed mainly toward external rewards [5]. In contrast to extrinsic values, intrinsic values are more related to psychological needs and fulfillment.

A few studies have explored and provided modified classifications of personal values based on early studies [4–5, 16–17]. Burroughs and Rindfleisch [19] conceptualized materialism as a self-centered value that is opposed to collective-oriented values like family, community ties, and religious fulfillment. Based on the studies of Schwartz [16–17], materialism, achievement, hedonism, and power can be categorized into the dimension of self-enhancement, whereas religiosity can be categorized as self-transcendence [19]. Grouzet et al. [20] provided a modified two-dimensional value structure considering that some specific values can be neither intrinsic nor extrinsic. For example, spirituality is not classified as intrinsic or extrinsic value. Spirituality is included in self-transcendence, in the opposite direction of physical self (i.e., hedonism) [20].

### Associations between personal values and happiness

Numerous studies have contrasted intrinsic and extrinsic values in terms of their associations with happiness. It has been widely observed that extrinsic values are negatively associated with happiness in Western as well as non-Western societies [8, 18]. In contrast with intrinsic goals like self-acceptance, extrinsic values of economic success, popularity, and image are adversely associated with happiness in Peru [8], China [12], South Korea [13], and Japan [21], as well as in Western societies, such as Germany and the United States [18]. A specific indicator of extrinsic values, viz. materialism, is also adversely associated with overall subjective well-being [6–7], satisfaction with life in family [22], and work [23] and positively correlated with depression and anxiety [19].

A few studies have investigated more diverse or specific personal values. Compared with materialism, which is a self-centered value and similar to the dimension of self-enhancement, collective-oriented values, such as family, community and religious values, appear to be beneficial for well-being [19]. Spirituality measured by religious values or practice is positively associated with subjective well-being [7, 19]. A longitudinal study has reported that prioritizing family over work and leisure results in higher life satisfaction [11]. Recent studies have also shown that prioritizing money more than time is adversely associated with happiness [9–10]. Although there are variations in terms of categorization of personal values, previous studies have provided quite consistent results showing that prioritizing extrinsic achievements, such as money, is adversely associated with subjective well-being in general.

Why are extrinsic or self-centered values adversely associated with happiness? On the one hand, it can be explained in that extrinsic values facilitate social comparison of oneself with others, which is harmful for subjective well-being. Extrinsic achievements are more easily compared with others than are intrinsic achievements, such as self-fulfillment or attachment. For example, people with high levels of materialism are more likely to compare themselves with others [24]. With greater social comparison, there is higher likelihood of frustration and dissatisfaction with individual achievements. People who prioritize extrinsic aspirations, such as power, money, or status, tend to have more difficulty of achieving and being satisfied with their goals.

On the other hand, extrinsic values can be harmful for interpersonal and social relationships. Pursuing material gains is negatively associated with quality of interpersonal relationships [25–26] and increases difficulty of achieving a family–work balance [27], which then decreases subjective well-being. People often need to decide whether they will spend time on social relationships or on extrinsic goals. People prioritizing extrinsic values are less likely to invest in social relationships, such as family and friends, which can decrease the quality of social relationships that is important for happiness. Recent studies have similarly argued that valuing money more than time may have deleterious impacts on social relationships [9–10]. These studies suggest that self-centered values or valuing self-enhancement is harmful for happiness, whereas collective-centered values or valuing social relationships is beneficial.

In this context, specific life domains might be differentially associated with happiness according to the attribute and nature of life domains. It is probable that prioritizing a specific life domain is negatively associated with happiness as the life domain is more based on self-centered value or self-interest. In contrast, we expect that life domains related to social relationships (alter-centered rather than self-centered) or self-transcendence are positively associated with happiness. In addition, life domains which have been classified as a same value category may have different effects on happiness depending on the degree to which they are self-centered value. For example, although health is conceptualized as intrinsic value [5, 20], it may have different meaning and effect for individuals compared with other intrinsic values or goals such as prioritizing family and friend. Prioritizing health can be self-centered propensity more than other intrinsic values such as prioritizing family. We classified the personal values regarding diverse life domains into four categories: prioritizing social relationships, extrinsic achievements, physical self, and spirituality, which reflects the different levels of self-centered propensity.

## Methods

### Data

Data were drawn from the Korean General Social Survey (KGSS) collected in 2007, 2008, and 2009. The KGSS is a nationally representative, cross-sectional survey conducted in South Korea [28]. The sampling method (i.e., multistage area proportional probability sampling), interview protocols, and data-processing procedures used for the KGSS conform to those used for the General Social Survey (GSS) conducted in the United States. Similar to the GSS, the KGSS includes special sets of questions every year in addition to core items, such as questions about socio-demographic factors. The KGSS in 2007–2009 included questions about personal values regarding life domains, happiness, and socio-demographic factors. Although the three years of data are not panel data, analyzing them as a pooled sample increases the statistical power for our analyses. The data were analyzed anonymously. The data for this study were made available by the Korean Social Science Data Archive (KOSSDA), Seoul National University, Seoul, South Korea.

### Measures

#### Happiness

Our dependent variable was subjective well-being (SWB), or happiness. Respondents were asked to rate their happiness via the following question: “When considering your life, how happy or unhappy are you overall?” The response categories of the 2007 and 2008 KGSS ranged from 1 (very happy) to 4 (not happy at all), whereas the happiness of 2009 was measured by a 5-point scale with a neutral category in the middle of the response categories. Due to this difference in response categories, we coded happiness as a binary variable in which two positive responses (i.e., very happy and happy) were assigned value 1 and the other responses were assigned value 0.

#### Personal values

To measure personal values regarding life domains, respondents were asked to choose two items as the first and second most important domain in life among the following 10 items: (1) leisure; (2) friends; (3) power; (4) neighbors; (5) health; (6) money; (7) educational attainment; (8) religion; (9) family; (10) work. We used a response for the first most important domain in life only for our analysis. We classified the responses into four categories: (1) social relationships; (2) extrinsic achievements; (3) physical self; and (4) spirituality. Social relationships included family, friends, and neighbors, and extrinsic achievements included leisure, power, money, educational attainment, and work. We included leisure in the category of extrinsic achievements because leisure can be considered an external reward related to self-interest. Physical self and spirituality were each represented by a single item (i.e., health and religion, respectively). Prioritizing physical self refers to placing importance on maintaining physical health and survival in the present study. In the analyses, the reference group of the variable was the respondents prioritizing social relationships. All categories of personal values were mutually exclusive.

#### Other covariates

Socio-demographic factors including gender, age, educational attainment, and marital status were measured. Gender was a binary variable with reference category of male (female = 1). Age was measured in years, and education attainment was classified into three categories: less than high school; high school graduates; college or more. The reference group for educational attainment in the analytic models was high school graduates. Marital status was measured by asking the respondents whether they were currently married, widowed, separated/divorced, or never married. The reference group for marital status in the analytical models was married.

We controlled for monthly household income and perceived social status as potential confounders of the association between personal values and happiness. Monthly household income was measured as a continuous variable in Korean 10,000 Won increments. We adjusted household income for the inflation rate across the three years of data, using the 2010 consumer price index [29]. After adjusting for the inflation rate, household income was log-transformed for the analyses because it was skewed. Perceived social status was measured with the question: “In our society, there are groups that tend to be positioned toward the top and those positioned toward the bottom. From the bottom (1) to the top (10), where would you put yourself on the scale?” Self-rated health was measured on a 5-point scale, and it was included in additional models examining the 2007 and 2009 data only because the 2008 KGSS did not include a self-rated health question.

### Analytical strategy

We used Poisson regression models with robust error variances for a binary outcome [30] given that our dependent variable (happiness) had high prevalence. Logistic regression results in misleading and overestimated odds ratios when it examines common outcomes whose incidence is higher than 10% [31]. Thus, relative risks of Poisson regression with a robust error variance would be appropriate for our dependent variable.

We conducted two sets of Poisson regression analyses to examine how personal values regarding the core life domains are associated with happiness. The first set comprised four models. Model 1 included personal values in life domains with year dummies only; Model 2 added the socio-demographic factors of gender, educational attainment, and marital status to Model 1. Model 3 added household income and perceived social status to examine whether personal values are associated with happiness even after controlling for these two variables. We analyzed Model 4, as a supplementary model, excluding the year 2009 (which had different response categories compared to other years) to check whether personal values remained associated with happiness.

We used the second set of Poisson regression models to examine the associations between personal values and happiness with age restriction and/or self-rated health as a covariate. We conducted supplementary analyses excluding respondents aged 60 years or older and controlling for self-rated health, measured in the 2007 and 2009 data.

## Results

### Sample characteristics

Table 1 presents the descriptive characteristics of the sample for the pooled data and for each survey year. For the total sample of pooled data, about 66% of respondents reported that they were happy overall; by year, 76.6%, 73.5%, and 49.7% reported being happy overall in 2007, 2008, and 2009, respectively. It is notable that the percentage of those who were happy in 2009 was lower than in the other two years. This is most likely because the 2009 survey used a 5-point scale to measure happiness, rather than the 4-point scale of the other years, leading to a substantial number of respondents (37.9%, 606 of 1,599) choosing the neutral category (which we coded as 0 = not happy).

**Table 1:** Descriptive statistics for all variables in the total sample and across survey years.

	Total		Year	
	(N = 4537)^a^	2007 (N = 1431)^a^	2008 (N = 1507)^a^	2009 (N = 1599)^a^
Happiness	0.660	0.766	0.735	0.497
	(0.474)	(0.423)	(0.441)	(0.500)
Personal values on life domains				
Social relationships	0.318	0.286	0.273	0.390
Extrinsic achievements	0.137	0.143	0.133	0.135
Physical self	0.507	0.533	0.565	0.430
Spirituality	0.038	0.038	0.029	0.045
Gender (female = 1)	0.533	0.539	0.541	0.519
Age	44.108	44.249	44.612	43.505
	(15.918)	(16.096)	(16.422)	(15.249)
Educational attainment				
Less than high school	0.214	0.237	0.222	0.187
High school graduate	0.300	0.285	0.301	0.314
College or over	0.485	0.478	0.478	0.499
Marital status				
Married	0.658	0.649	0.656	0.669
Widowed	0.083	0.091	0.094	0.066
Divorced/separated	0.036	0.038	0.035	0.034
Never married	0.223	0.223	0.215	0.231
Monthly household income (logged)	4.584	4.524	4.583	4.640
	(0.936)	(0.959)	(0.927)	(0.929)
Perceived social status	4.581	4.612	4.627	4.509
	(1.621)	(1.631)	(1.658)	(1.574)
Year				
2007	0.315			
2008	0.332			
2009	0.352			

^a^ The number of cases may vary due to missing data.

Remarks: Means/proportions are presented. Numbers in parentheses are standard deviations. Standard deviations of binary variables are excluded.

In terms of personal values regarding life domains, 50.7% of respondents considered health to be the most important domain in life; 31.8% chose family, friends, or neighbors; 13.7% chose extrinsic achievements including money, power, educational attainment, work, and leisure; and 3.8% chose religion. Across years, a higher percentage of respondents in 2009 prioritized social relationships than in 2007 and 2008 (i.e., 28.6% in 2007, 27.3% in 2008, and 38% in 2009). However, the percentages of those prioritizing extrinsic achievements were consistent across years (i.e., 14.3% in 2007, 13.3% in 2008, and 13.5% in 2009).

Table 2 summarizes the descriptive statistics across personal values and provides the results of the Chi-squared or analysis of variance (ANOVA) tests comparing proportions or means of variables across the categories of personal values. For happiness, 81.2% of those who prioritized religion the highest answered that they were happy overall, which was the highest percentage observed. In contrast, about 54.7% of respondents prioritizing extrinsic achievements answered that they were happy, which was the lowest observed value. Among respondents prioritizing social relationships and health, 70.7% and 65% answered that they were happy, respectively. Bivariate statistics comparing distributions or means of the variables depending on personal values showed that all variables had significant differences depending on personal values regarding life domains.

**Table 2:** Descriptive statistics for all variables across values on life domains and the results of Chi-squared/ANOVA tests comparing variables across the life domains.

	Social relationships (N = 1440)^a^	Extrinsic achievements (N = 620)^a^	Physical self (N = 2297)^a^	Spirituality (N = 171)^a^	Chi-square/ ANOVA test
Happiness	0.707	0.547	0.650	0.812	***
Gender (female = 1)	0.520	0.444	0.558	0.614	***
Age	40.180	38.388	47.962	45.696	***
	(14.854)	(15.236)	(15.627)	(16.739)	
Educational attainment^b^					***
Less than high school	0.216	0.091	0.661	0.033	
High school graduate	0.298	0.138	0.541	0.023	
College or over	0.376	0.157	0.419	0.049	
Marital status^b^					***
Married	0.319	0.106	0.534	0.041	
Widowed	0.197	0.082	0.684	0.037	
Divorced/separated	0.236	0.130	0.609	0.025	
Never married	0.375	0.250	0.347	0.029	
Monthly household income (logged)	4.707	4.684	4.492	4.475	***
	(0.819)	(0.898)	(0.998)	(0.962)	
Perceived social status	4.786	4.592	4.435	4.805	***
	(1.554)	(1.619)	(1.641)	(1.674)	
Year^b^					
2007	0.286	0.143	0.533	0.039	
2008	0.273	0.133	0.565	0.029	
2009	0.390	0.135	0.430	0.045	

^a^The number of cases may vary due to missing data.

^b^ The row sum of proportions is 1.

+p<0.10.

*p<0.05.

**p<0.01.

***p<0.001.

Remarks: Numbers in parentheses are standard deviations. Standard deviations of binary variables are excluded. 8 respondents who did not respond to the question about life values were excluded.

**Fig 1:**
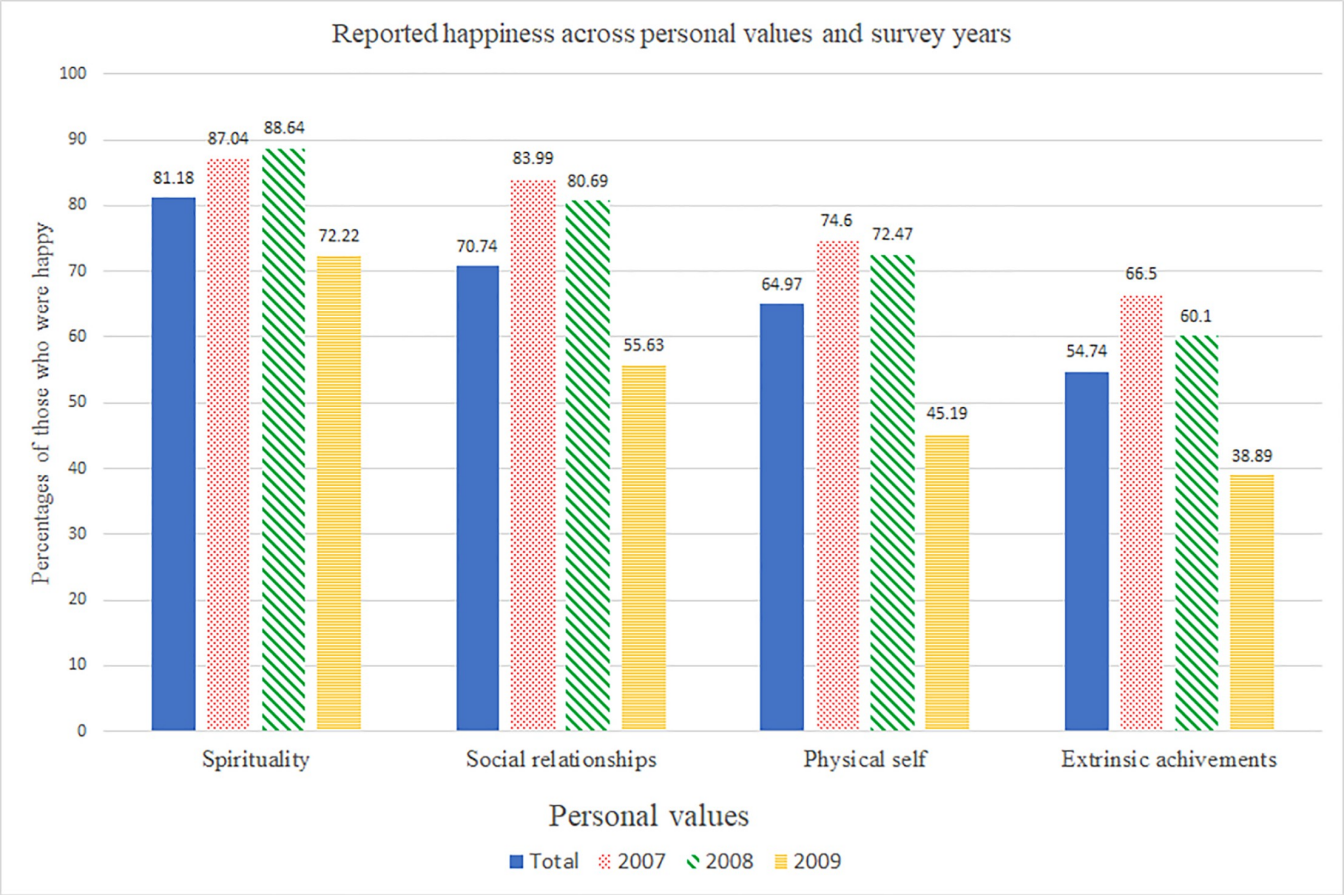
Percentages of respondents who reported being happy across valued life domains and survey years.

Fig 1 shows the percentages of those who were happy across personal values and survey years. Reported happiness varied across the survey years, but we also observed consistent patterns linking personal values with happiness (Fig 1). Respondents prioritizing spirituality and social relationships showed higher percentages of happiness than the others. Respondents who valued extrinsic achievements showed the lowest percentages of happiness across all years. Note that percentages of reported happiness were lower across all personal values in 2009 than in 2007 and 2008 due to the different response categories used in 2009. It would be also possible that the percentage of reported happiness in 2009 was dropped because the data were collected after the global economic crisis in 2008.

### Poisson regression models

Table 3 summaries the results of Poisson regression models with robust error variances examining associations between personal values and happiness. Models 1, 2, and 3 showed that all categories of personal values had significant relationships with happiness. Compared with those prioritizing social relationships, respondents valuing extrinsic achievements and health had lower likelihoods of being happy, whereas those prioritizing religion were happier than the reference group. The categories of personal values were significant, even after controlling for household income and perceived social status, as well as socio-demographic factors in Model 3. Relationships between personal values and happiness are also shown in Model 4, excluding the 2009 data. Prioritizing extrinsic achievements was again adversely associated with happiness. Respondents prioritizing health were also less likely to report happiness than those prioritizing social relationships. One notable difference between the results of Model 4 and the other models was that prioritizing religion was not significant in Model 4 from which the 2009 data were excluded.

**Table 3:** Poisson regression analyses with robust error variances examining associations between personal values on life domains and happiness (relative risks).

	Model 1		Model 2		Model 3		Model 4	
Personal values on life domain (social relationships = referent)								
Extrinsic achievements	0.748	***	0.770	***	0.786	***	0.805	***
Physical self	0.871	***	0.919	***	0.930	**	0.939	**
Spirituality	1.141	**	1.126	**	1.136	**	1.022	
Gender (female = 1)			1.048	*	1.044	*	1.061	**
Age			0.995	***	0.995	***	0.998	*
Educational attainment (High school graduate = referent)								
Less than high school			0.926	+	1.003		0.964	
College or over			1.124	***	1.039		1.016	
Marital status (Married = referent)								
Widowed			0.825	**	0.887	*	0.816	**
Divorced/separated			0.600	***	0.662	***	0.686	***
Never married			0.792	***	0.805	***	0.798	***
Monthly household income(logged)					1.059	***	1.059	***
Perceived social status					1.082	***	1.068	***
Year (2007 = referent)								
2008	0.963	+	0.960	*	0.959	*	0.957	*
2009	0.636	***	0.632	***	0.639	***		
Observations	4495		4480		4340		2796	

+p<0.10.

*p<0.05.

**p<0.01.

***p<0.001.

Gender, age, and marital status were significantly associated with happiness in Models 3 and 4. Females tended to be happier than males. Married respondents tended to be happier than unmarried respondents. The likelihood of being happy decreased as age increased. In terms of socio-economic status, both household income and perceived social status were significantly associated with happiness. The likelihood of being happy increased as household income and perceived social status increased.

Table 4 presents the results of the additional models showing the associations between personal values regarding life domains and happiness with age restriction and/or controlling for self-rated health. Model 1 excluded respondents aged 60 or older from the total sample, and Model 2 included self-rated health as a control variable in the 2007 and 2009 data. In Model 3, the age restriction was also applied with self-rated health, so Models 2 and 3 included only 2007 and 2009 data because information on self-rated health was not collected in 2008. As listed in Table 4, all categories of personal value were significantly associated with happiness, regardless of whether age and/or data restrictions were imposed. Compared with prioritizing social relationships, prioritizing extrinsic achievements was adversely associated with happiness even after controlling for self-rated health and excluding those aged 60 years or older in Model 3. Prioritizing physical self was, however, marginally significant in Model 3 with self-rated health and the age restriction. Respondents who prioritized religion were most likely to be happy.

**Table 4:** Poisson regression analyses with robust error variances with age restriction (excluding respondents aged 60 years or older) and/or controlling for self-rated health in 2007 and 2009 surveys (relative risks).

	Model 1		Model 2		Model 3	
Personal values on life domain (social relationships = referent)						
Extrinsic achievements	0.805	***	0.807	***	0.838	***
Physical self	0.946	*	0.924	**	0.943	+
Spirituality	1.164	***	1.151	*	1.181	**
Gender (female = 1)	1.037	+	1.074	**	1.070	*
Age	0.993	***	0.997	*	0.994	**
Educational attainment (High school graduate = referent)						
Less than high school	0.996		1.096	+	1.123	+
College or over	1.053	*	1.053		1.061	+
Marital status (Married = referent)						
Widowed	0.869		0.997		1.002	
Divorced/separated	0.688	***	0.763	*	0.818	+
Never married	0.778	***	0.812	***	0.777	***
Monthly household income(logged)	1.061	***	1.040	*	1.050	*
Perceived social status	1.076	***	1.074	***	1.068	***
Self-rated health (2007 & 2009 data only)			1.164	***	1.177	***
Year (2007 = referent)						
2008	0.969					
2009	0.661	***	0.639	***	0.666	***
observations	3552		2901		2398	

+p<0.10.

*p<0.05.

**p<0.01.

***p<0.001.

Remarks: Model 1 = Total sample with age restriction; Model 2 = Only 2007 and 2009 data including self-rated health; Model 3 = Model 2 + age restriction

In addition, we conducted two supplementary sets of Poisson and multiple linear regression analyses as sensitivity analyses. The supplementary sets of Poisson regression models included personal values in life domains with leisure as a single category. It would be worth examining leisure separately because it can be closer to hedonism compared with the other extrinsic achievements. The supplementary sets of multiple linear regression models included the dependent variable as a continuous variable by multiplying the 4-point scale by 5 and multiplying the 5-point scale by 4 so that we could examine the continuous dependent variable with a 20-point scale and test if it had consistent results with Poisson regression analyses examining the dichotomized dependent variable.

S1 Table presents the results of Poisson regression models corresponding to the analytical models of Table 3. Prioritizing leisure was negatively associated with happiness (S1 Table). Other personal value variables had consistent results with the findings in Table 3. S2 Table presents the results of selected four multiple regression models due to word limitation. However, we had consistent results with our findings across all corresponding models in terms of effects and significances of personal value variables. Only one difference from the results of the Poisson regression analyses was that spirituality was still significant when the 2009 data were excluded as presented in Models 3 and 4 (S2 Table).

## Discussion

Our findings showed that there were significant associations between personal values regarding life domains and happiness. Prioritizing social relationships, including family, friends, and neighbors, was associated with a greater likelihood of happiness, whereas prioritizing extrinsic achievements, such as money and power, or physical self (i.e., health) was adversely associated with happiness. Although prioritizing spirituality (i.e., religion) was not significant when excluding the 2009 data, it was significantly and positively associated with happiness in the models when the age restriction was employed, or with self-rated health, as well as for the total sample. Respondents prioritizing religion were most likely to report happiness, whereas respondents prioritizing extrinsic achievements were the least likely. A significant difference between prioritizing extrinsic achievements and prioritizing health persisted in our supplementary models (S3 Table), in which extrinsic achievements was set as the referent category. Thus, we found that the rank order of happiness across personal values regarding life domains, from the highest to lowest likelihood, was spirituality, social relationships, physical self, and extrinsic achievements. Although previous studies have consistently shown that religious affiliation is positively associated with happiness [32–33], our findings have newly shown that respondents prioritizing religion are most likely to be happy than others.

The current findings support previous studies showing adverse associations between extrinsic, self-enhancement, or self-centered values and happiness [6–7, 18]. Adverse associations between prioritizing extrinsic achievements and happiness can be explained in that extrinsic values facilitate social comparisons [24] and decrease quality of interpersonal relationships [25–26]. Prioritizing family over work and leisure enhances life satisfaction by increasing family satisfaction [11]. Recent studies [9–10] have similarly suggested that prioritizing time over money is beneficial for happiness via increasing the quality of social relationships. It is likely that people who consider extrinsic achievements as the most important thing in life are less likely to be satisfied with their current achievements and less likely to invest in social relationships, such as family and friends.

We also found that prioritizing social relationships is important for happiness and more beneficial than valuing extrinsic achievements or even physical self. This finding is consistent with a previous study showing that collective-centered values are more beneficial for well-being than are self-centered values [19]. Respondents prioritizing social relationships may tend to have higher quality of social relationships than those who value extrinsic rewards or egos (i.e., physical self). Additionally, spirituality, which can be classified into the dimension of self-transcendence or selflessness, is even more beneficial for happiness than is prioritizing social relationships. Psychological fulfillment through religion can be beneficial for happiness. Spirituality also may increase happiness in that it promotes a non-materialistic attitude toward life and decreases social comparison [7].

In sum, our findings showed how level of happiness is ranked according to the priority assigned to different personal values, with the highest level of happiness associated with spirituality, followed by social relationships, physical self, and (lastly) extrinsic achievements. This suggests that a greater propensity toward being self-centered is inversely associated with happiness. Among the four personal values, prioritizing extrinsic achievements can be considered as the strongest self-centered propensity whereas spirituality is the least self-centered propensity in that it could be categorized as self-transcendence [19]. Physical self might be intermediate between prioritizing extrinsic achievements and prioritizing social relationships. Although health is often conceptualized as an intrinsic value [5, 20], prioritizing health might be more self-centered than prioritizing social relationships.

Some limitations of this study merit consideration. First, the data are cross-sectional and therefore we are limited in our ability to draw causal inferences. For example, it is possible that people who are unhappy with their social relationships are more likely to direct their attention toward earning the respect of others by seeking status, wealth, and power (reverse causality). In this scenario, individuals who are currently focused on prioritizing extrinsic achievements might not achieve happiness by being counseled to redirect their attention to their social relationships. Second, we used only three-year data of the KGSS (2007, 2008, and 2009) although the KGSS has been collected annually from 2003 to 2014, and biannually from 2014. The KGSS included both the personal value and happiness questions analyzed in the study for the three-year period only.

Third, we should be cautious about generalizing the findings about spirituality. Prioritizing religion was not significant in Model 4, shown in Table 3, from which the 2009 data were excluded, although it was significant in the other models overall even with age restriction or with controlling for self-rated health, as shown in Table 4. The lack of significance of spirituality when excluding the 2009 data might be due to the resulting decrease in statistical power. Compared with the 2007 and 2008 surveys, a slightly higher percentage of respondents chose religion as the most important domain in 2009 (i.e., 3.8% in 2007, 2.9% in 2008, and 4.5% in 2009). Only 3.38% of respondents (i.e., 99 of 2,933) chose religion as the most important domain after excluding the 2009 data, which might decrease statistical power. Further studies on associations between spirituality and happiness are needed to clarify these relationships. Finally, although it is reasonable to classify the 10 investigated life domains into four categories, more diverse classifications are needed in further studies. For example, we could not categorize neighbors as a separate category from social relationships because of the limited number of respondents who chose neighbors (i.e., 0.95% of respondents).

In spite of the limitations, this study extends previous knowledge about personal values and happiness by examining individual priorities for specific life domains and their impacts on happiness. Happiness may increase as individuals prioritize alters over egos, and egos over extrinsic rewards, which provides an ironic, but important implications about happiness in the individualistic and materialistic world.

## Supporting information

S1 Table Poisson regression analyses with robust error variances including prioritizing leisure as a single category (relative risks). (DOCX)

S2 Table Multiple regression analyses examining associations between personal values on life domains and happiness. (DOCX)

S3 Table Poisson regression analyses with robust error variances including extrinsic achievements as a referent category (relative risks). (DOCX)
