# Supplementary material for: The keys to happiness: Associations between personal values regarding core life domains and happiness in South Korea
Source: PLoS One. 2019 Jan 9;14(1):e0209821. doi: 10.1371/journal.pone.0209821 (PMC6326475; doi:10.1371/journal.pone.0209821)
Supplement: S1 Table — (DOCX) [file pone.0209821.s001.docx]

S1 Table. Poisson regression analyses with robust error variances including prioritizing leisure as a single category (relative risks)

|  | Model 1 |  | Model 2 |  | Model 3 |  | Model 4 |  |
| --- | --- | --- | --- | --- | --- | --- | --- | --- |
| Personal values on life domain (social relationships=referent) |  |  |  |  |  |  |  |  |
| Extrinsic achievements except leisure | 0.739 | *** | 0.762 | *** | 0.782 | *** | 0.788 | *** |
| Leisure | 0.792 | ** | 0.806 | ** | 0.805 | ** | 0.879 | + |
| Physical self | 0.871 | *** | 0.919 | *** | 0.930 | ** | 0.939 | ** |
| Spirituality | 1.141 | ** | 1.126 | ** | 1.136 | ** | 1.022 |  |
| Gender (female=1) |  |  | 1.048 | * | 1.043 | * | 1.060 | ** |
| Age |  |  | 0.995 | *** | 0.995 | *** | 0.998 | * |
| Educational attainment (High school graduate=referent) |  |  |  |  |  |  |  |  |
| Less than high school |  |  | 0.926 | + | 1.003 |  | 0.964 |  |
| College or over |  |  | 1.123 | *** | 1.039 |  | 1.015 |  |
| Marital status (Married=referent) |  |  |  |  |  |  |  |  |
| Widowed |  |  | 0.826 | ** | 0.887 | * | 0.817 | ** |
| Divorced/separated |  |  | 0.601 | *** | 0.662 | *** | 0.687 | *** |
| Never married |  |  | 0.791 | *** | 0.805 | *** | 0.797 | *** |
| Monthly household income(logged) |  |  |  |  | 1.059 | *** | 1.059 | *** |
| Perceived social status |  |  |  |  | 1.082 | *** | 1.068 | *** |
| Year (2007=referent) |  |  |  |  |  |  |  |  |
| 2008 | 0.963 | + | 0.961 | + | 0.959 | * | 0.958 | * |
| 2009 | 0.637 | *** | 0.632 | *** | 0.639 | *** |  |  |
| observations | 4495 |  | 4480 |  | 4340 |  | 2796 |  |
| +p<0.10, *p<0.05, **p<0.01, ***p<0.001 | |  |  |  |  |  |  |  |

Remark: Model 4 included the 2007 and 2008 data only.
