# Supplementary material for: The keys to happiness: Associations between personal values regarding core life domains and happiness in South Korea
Source: PLoS One. 2019 Jan 9;14(1):e0209821. doi: 10.1371/journal.pone.0209821 (PMC6326475; doi:10.1371/journal.pone.0209821)
Supplement: S2 Table — (DOCX) [file pone.0209821.s002.docx]

S2 Table. Multiple regression analyses examining associations between personal values on life domains and happiness

|  | Model 1 |  | Model 2 |  | Model 3 |  | Model 4 |  |
| --- | --- | --- | --- | --- | --- | --- | --- | --- |
| Personal values on life domain (social relationships=referent) |  |  |  |  |  |  |  |  |
| Extrinsic achievements including leisure | -1.139 | *** |  |  | -1.119 | *** |  |  |
|  | (0.164) |  |  |  | (0.203) |  |  |  |
| Extrinsic achievements except leisure |  |  | -1.186 | *** |  |  | -1.251 | *** |
|  |  |  | (0.175) |  |  |  | (0.218) |  |
| Leisure |  |  | -0.899 | * |  |  | -0.524 |  |
|  |  |  | (0.347) |  |  |  | (0.407) |  |
| Physical self | -0.449 | *** | -0.449 | *** | -0.486 | ** | -0.489 | ** |
|  | (0.117) |  | (0.117) |  | (0.146) |  | (0.146) |  |
| Spirituality | 1.447 | *** | 1.447 | *** | 1.075 | ** | 1.075 | ** |
|  | (0.276) |  | (0.276) |  | (0.354) |  | (0.354) |  |
| Gender (female=1) | 0.029 |  | 0.029 |  | 0.065 |  | 0.062 |  |
|  | (0.105) |  | (0.105) |  | (0.128) |  | (0.128) |  |
| Age | -0.026 | *** | -0.026 | *** | -0.020 | ** | -0.020 | ** |
|  | (0.005) |  | (0.005) |  | (0.006) |  | (0.006) |  |
| Educational attainment (High school graduate=referent) |  |  |  |  |  |  |  |  |
| Less than high school | 0.092 |  | 0.093 |  | -0.066 |  | -0.063 |  |
|  | (0.174) |  | (0.174) |  | (0.210) |  | (0.210) |  |
| College or over | 0.142 |  | 0.141 |  | 0.194 |  | 0.190 |  |
|  | (0.125) |  | (0.125) |  | (0.154) |  | (0.256) |  |
| Marital status (Married=referent) |  |  |  |  |  |  |  |  |
| Widowed | -0.553 | * | -0.550 | * | -1.038 | *** | -1.032 | *** |
|  | (0.222) |  | (0.222) |  | (0.256) |  | (0.256) |  |
| Divorced/separated | -1.893 | *** | -1.888 | *** | -1.962 | *** | -1.955 | *** |
|  | (0.278) |  | (0.278) |  | (0.333) |  | (0.333) |  |
| Never married | -1.321 | *** | -1.325 | *** | -1.481 | *** | -1.493 | *** |
|  | (0.156) |  | (0.156) |  | (0.190) |  | (0.190) |  |
| Monthly household income(logged) | 0.291 | *** | 0.291 | *** | 0.250 | ** | 0.250 | ** |
|  | (0.062) |  | (0.062) |  | (0.077) |  | (0.077) |  |
| Perceived social status | 0.423 | *** | 0.422 | *** | 0.410 | *** | 0.408 | *** |
|  | (0.036) |  | (0.036) |  | (0.044) |  | (0.044) |  |
| Year (2007=referent) |  |  |  |  |  |  |  |  |
| 2008 | -0.149 |  | -0.147 |  | -0.153 |  | -0.147 |  |
|  | (0.125) |  | (0.125) |  | (0.122) |  | (0.122) |  |
| 2009 | -0.583 | *** | -0.581 | *** |  |  |  |  |
|  | (0.124) |  | (0.124) |  |  |  |  |  |
| Constant | 12.973 | *** | 12.978 | *** | 13.064 | *** | 13.079 | *** |
|  | (0.414) |  | (0.414) |  | (0.504) |  | (0.504) |  |
| N | 4340 |  | 4340 |  | 2796 |  | 2796 |  |
| F | 47.84 | *** | 44.69 | *** | 36.52 | *** | 34.13 | *** |
| R^2^ | 0.134 |  | 0.134 |  | 0.146 |  | 0.147 |  |
| +p<0.10, *p<0.05, **p<0.01, ***p<0.001 | |  |  |  |  |  |  |  |

Remarks: Unstandardized coefficients (standard errors) are presented. Models 3 and 4 included the 2007 and 2008 data only.
