# Supplementary material for: The keys to happiness: Associations between personal values regarding core life domains and happiness in South Korea
Source: PLoS One. 2019 Jan 9;14(1):e0209821. doi: 10.1371/journal.pone.0209821 (PMC6326475; doi:10.1371/journal.pone.0209821)
Supplement: S3 Table — (DOCX) [file pone.0209821.s003.docx]

S3 Table. Poisson regression analyses with robust error variances including extrinsic achievements as a referent category (relative risks)

|  | Model 1 |  | Model 2 |  |
| --- | --- | --- | --- | --- |
| Personal values on life domain (Extrinsic achievements=referent) |  |  |  |  |
| Social relationships | 1.272 | *** | 1.242 | *** |
| Physical self | 1.183 | *** | 1.166 | *** |
| Spirituality | 1.445 | *** | 1.269 | *** |
| Gender (female=1) | 1.044 | * | 1.061 | ** |
| Age | 0.995 | *** | 0.998 | * |
| Educational attainment (High school graduate=referent) |  |  |  |  |
| Less than high school | 1.003 |  | 0.964 |  |
| College or over | 1.039 |  | 1.016 |  |
| Marital status (Married=referent) |  |  |  |  |
| Widowed | 0.887 | * | 0.816 | ** |
| Divorced/separated | 0.662 | *** | 0.686 | *** |
| Never married | 0.805 | *** | 0.798 | *** |
| Monthly household income(logged) | 1.059 | *** | 1.059 | *** |
| Perceived social status | 1.082 | *** | 1.068 | *** |
| Year (2007=referent) |  |  |  |  |
| 2008 | 0.959 | * | 0.957 | * |
| 2009 | 0.639 | *** |  |  |
| observations | 4340 |  | 2796 |  |
| +p<0.10, *p<0.05, **p<0.01, ***p<0.001 |  |  |  |  |

Remark: Models 1 and 2 are comparable to Models 3 and 4 in Table 3.
